# Life Cycle Inventory and Assessment Datasets on the Operational Sustainability of the Ammonia Process

**DOI:** 10.1016/j.dib.2020.105593

**Published:** 2020-04-22

**Authors:** Sherard Sadeek, Thérèse Lee Chan, Rukam Ramdath, Aqueel Rajkumar, Miao Guo, Keeran Ward

**Affiliations:** a Department of Chemical Engineering, University of the West Indies, St. Augustine, Trinidad and Tobago; b Department of Engineering, King’s College London, Strand Campus, United Kingdom, WC2R 2LS

**Keywords:** Ammonia Process, Sustainability, Life Cycle Assessment, Carbon Capture Utilization, KBR, Process design

## Abstract

This article presents data affiliated with life cycle inventories, environmental impact and operational sustainability used in, the influence of raw material availability and utility power consumption on the sustainability of the ammonia process [1]. Scenario specific operating conditions were used to simulate the ammonia process based on unique constraints occurring within the Trinidad and Tobago energy sector. The data was collected using AspenⓇ Plus simulations and validated against plant operating data. The data consists of an economic cost evaluation as well as environmental impact using the CML-IA Baseline midpoint approach. The data was derived from life cycle inventories aligned to input/output material and energy flows within the ammonia process as well as life cycle assessment databases utilizing Ecoinvent v3.4. The data can be applied to the wider ammonia supply chain, aiding in achieving greater sustainable development within ammonia-based process industries.

Specifications table

**Table:** 

Subject	Chemical Engineering- Process Chemistry and Technology
Specific subject area	Life Cycle Assessment (LCA)
Type of data	Tables/Figures
How data were acquired	Mass and energy flows were collated from Aspen® Plus simulations validated against operating data derived from an ammonia plant within Trinidad and Tobago. For LCA, material flows were aligned to Ecoinvent V3.4 LCA databases. For economic assessment, operational costs were estimated from literature.
Data format	Raw/Analysed.
Parameters for data collection	Raw material, product and waste material flows, heat integration energy flows, utility steam and cooling water flows.
Description of data collection	Input/output flows procured from Aspen Plus simulations. LCA databases procured from Ecoinvent v3.4.
Data source location	Point Lisas, Trinidad and Tobago.
Data accessibility	Repository Name: Mendeley Data; DOI: 10.17632/7krrvxtsmm.2
	URL: https://data.mendeley.com/datasets/7krrvxtsmm/2
Related research article	Sherard Sadeek, Thérèse Lee Chan, Rukam Ramdath, Aqueel Rajkumar, Miao Guo and Keeran Ward, *The Influence of Raw Material Availability and Utility Power Consumption on the Sustainability of the Ammonia Process*, Chemical Engineering Research and Design, DOI:
	https://doi.org/10.1016/j.cherd.2020.03.020.

## Value of the data

• The data available gives insights into the sustainable assessment of operational ammonia plants and the impact of operating conditions on reducing environmental burden.
• The data can be used within the ammonia supply chain demand such as the fertilizer industry, to further investigate sustainable operations.
• The data can be easily compared with other ammonia process technologies to understand how operational efficiency can improve overall sustainability.

## 1 Data

The data presented here illustrate the datasets utilized from Ecoinvent Databases for the ammonia process system boundary definition as well as raw and processed data (available through supplementary files at http://dx.doi.org/10.17632/7krrvxtsmm.2) aligned to input-output flows developed using process modelling.

## 2 Experimental Design, Materials and Methods

### 2.1 Aspen Plus Simulation

The ammonia process was simulated using Aspen® Plus and validated against plant operating data. The Front End, Back End and Ammonia Refrigeration sections of the process were simulated using the Peng-Robinson thermodynamic fluid package. For carbon dioxide (CO_2_) removal, the Electrolyte non-random two-liquid – Redlich-Kwong and Perturbed-chain statistical associating fluid theory Equations of state were employed to simulate the CO_2_ capture using proprietary solvents.

In the front-end section of the process, hydrogen is produced in two stages. In the primary reforming step, compressed natural gas is mixed with steam and converted to hydrogen and carbon oxides (mainly CO and CO_2_). In secondary reforming, the reformed gas stream is mixed with air stoichiometrically to maintain the required amount of nitrogen required for ammonia synthesis, and hydrogen is produced whereby heat generated within the partial oxidation of methane facilitates the steam reforming and water gas shift reactions. All reforming reactions were modelled using equilibrium reactors with set approach temperatures. Waste heat recovery was considered downstream the hydrogen generation unit through high-pressure (HP) steam generation. Finally, CO is converted to CO_2_ in two stages through low and high temperature water shift conversion. These reactions were modelled using a R-STOIC reactor with set conversions.

From the front-end section of the plant, reformed gas is further cooled through heat recovery and sent to the CO_2_ removal section using proprietary solvent scrubbing. Both absorption and stripping processes were modelled as equilibrium RADFRAC applications and the equilibrium constants within the Aspen Plus library were used. The CO_2_ was removed and sent to the low-pressure flash section and the lean solvent was recycled to the absorber.

In the back-end section of the plant, methanation was considered whereby residual carbon oxides were converted to methane using hydrogen. Methanation reactions were modelled using a R-STOIC reactor with 100% conversion. The effluent was flashed and mixed with recycle hydrogen from the purge gas recovery unit and recycle gas from the refrigeration section, and sent to the ammonia synthesis converter. The ammonia synthesis converter was modelled as four equilibrium reactors (to represent 4 beds) and three heat exchangers with set approach temperatures and duties.

The synthesis gas was first preheated and sent to each bed whereby the heat of reaction was removed through heat recovery. The discharge was further cooled in the ammonia refrigeration section, whereby the produced ammonia was removed using cold ammonia gas recovered from purge gas as well as cold liquid ammonia product in a counter-current heat exchange process. This was simulated using a series of shell and tube heat exchangers, exchangers, flash drums and compressors. Detailed process descriptions and simulation assumptions are available [1].

### 2.2 Scenario Descriptions

Four (4) scenarios were developed based on current operational issues facing traditional ammonia processing within Trinidad and Tobago; Scenario 1- Availability of Raw Material, Scenario 2- Optimization of Purge Gas Recovery, Scenario 3-Optimization of Ammonia Synthesis, and Scenario 4- Utility Optimization. Carbon capture and Utilization (CCU) was also examined within Scenario 1 in an effort to investigate potential avenues for reduced environmental burden. Raw input/output material flows as well as energy integration were collated and normalized to the functional unit- 1000kg/h ammonia, forming the basis of life cycle inventories (LCI) for each scenario. Detailed scenario descriptions and objectives are available [1].

### 2.3 Economic Evaluation

Operational costs attributed to natural gas utilization, cooling water, steam generation, demineralized water and electricity as well as ammonia product revenue were estimated for each simulation using cost data supplied by market trends as well as literature [3], [4], [5], [6], [7]. Operational profit and Incremental savings were calculated following Eqs. 1 and 2:

(1) OperationalProfit=Revenue(sales)−OperationalCost

(2) $$Incremental\;Savings={\left[Operational\;Profit\right]}_{Base\;case}-{\left[Operational\;Profit\right]}_{Current\;case}$$

### 2.4 LCA Methodology

**Fig. 1:**
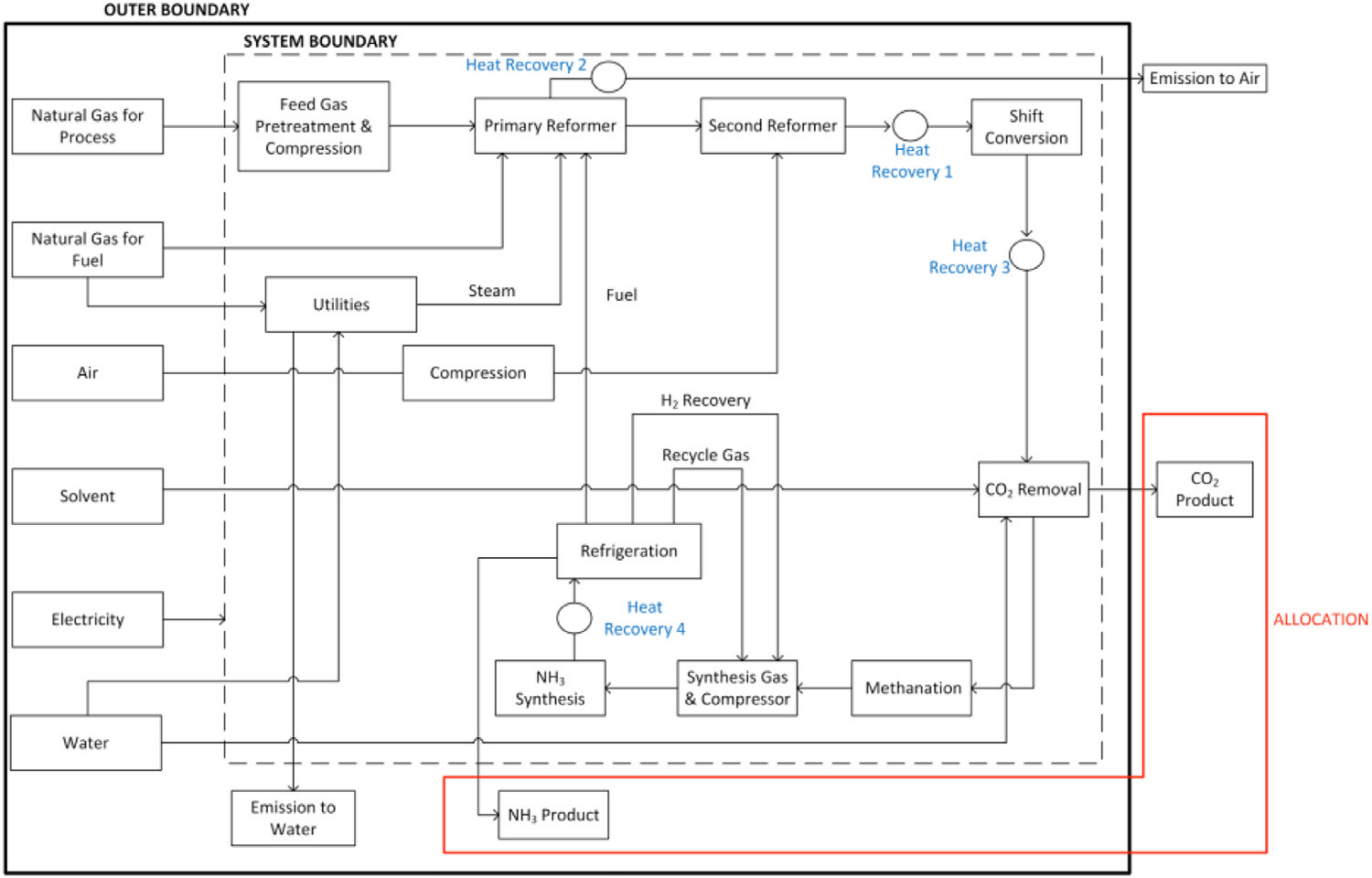
System Boundary Definition for the Ammonia Process.

The defined system boundary for the ammonia process is given in Fig. 1. Table 1 gives the LCA datasets used within the Ecoinvent v3.4 database for various material flows. Scenario specific LCI was aligned to each LCA datasets and used to investigate the environmental impact of the ammonia process across eleven (11) environmental impact categories (Table 2) using the CML-IA baseline Life Cycle Impact Assessment (LCIA) midpoint methodology [8]. In an effort to judge the sensitivity of the LCA results to the LCIA methodology, the ReCiPe Endpoint hierarchist V1.13 (Table 3) was used. An economic allocation was utilized based on the cost prices of both ammonia and CO_2_ to illustrate the effects of carbon capture and utilization as an environmental benefit for the ammonia process. Detailed analysis of the system boundary and relevant assumptions are given [1].

**Table 1:** LCA Datasets Retrieved from the Ecoinvent v3.4 Database.

Input-Output Flow	Technology Involved	Description in Ecoinvent
Heating	Natural Gas Combustion	Heat, district or industrial, natural gas {RoW}| heat production, natural gas, at industrial furnace >100kW | Alloc Def, U
Process Water	Deionization using Tap Water	Water, deionized, from tap water, at user {RoW}| production | Alloc Def, U
Natural Gas Feedstock	Natural Gas – Volume Basis	Natural gas, low pressure {RoW}| market for | Alloc Def, U
Electricity	Derived from Natural Gas Combustion	Electricity, high voltage {RoW}| electricity production, natural gas, 10MW | Alloc Def, U

**Table 2:** List of Impact Categories established using the CML-IA Baseline Midpoint LCIA Characterization Method [2].

Impact Group	Impact Category	Reference Unit
Acidification	Acidification Potential	kg SO_2_ eq
Climate Change	Global Warming Potential (GWP100a)	kg CO_2_ eq
Abiotic Resources	Depletion of Abiotic Resources- Elemental Reserves	kg Sb eq
	Depletion of Abiotic Resources- Fossil Fuels	MJ
Ecotoxicity	Freshwater Aquatic Ecotoxicity	kg 1,4-DB eq
	Marine Aquatic Ecotoxicity	kg 1,4-DB eq
	Terrestrial Ecotoxicity	kg 1,4-DB eq
Eutrophication	Eutrophication	kg PO_4_ eq
Human Toxicity	Human Toxicity	kg 1,4-DB eq
Ozone Layer Depletion	Ozone Layer Depletion	kg CFC-11 eq
Photochemical Oxidation	Photochemical Oxidation	kg C_2_H_4_ eq

**Table 3:** List of Impact Categories established using the ReCiPe Endpoint Hierarchist V1.13 LCIA Characterization Method.

Impact Group	Impact Category	Reference Unit
Acidification	Terrestrial Acidification	species/yr
Climate Change	Climate Change Human Health	DALY
	Climate Change Ecosystems	species/yr
Abiotic Resources	Metal Depletion	$
	Fossil Depletion	$
Ecotoxicity	Freshwater Ecotoxicity	species/yr
	Marine Ecotoxicity	species/yr
	Terrestrial Ecotoxicity	species/yr
Eutrophication	Freshwater	species/yr
Human Toxicity	Human Toxicity	DALY
Ozone Layer Depletion	Ozone Depletion	DALY
Ionizing Radiation	Ionizing Radiation	DALY
Land Use	Urban Land Occupation	species/yr
	Natural Land Transformation	species/yr
	Agricultural Land Occupation	species/yr
Particulate Matter	Particulate Matter Formation	DALY
Photochemical Oxidation	Photochemical Oxidant Formation	DALY
